# Facilitators and barriers in using comics to support family caregivers of patients receiving palliative care at home: A qualitative study

**DOI:** 10.1177/02692163221093513

**Published:** 2022-05-03

**Authors:** Maaike M Haan, Jelle LP van Gurp, Marjan Knippenberg, Gert Olthuis

**Affiliations:** Radboud University Medical Center, Radboud Institute for Health Sciences, IQ healthcare, Nijmegen, The Netherlands

**Keywords:** Family caregivers, informal care, graphic novels as topic, comic art, palliative care, education, arts-based research

## Abstract

**Background:**

Family caregiving at home is highly important for people receiving palliative treatment, but also a complex experience, subject to implicit social expectations. This study empirically explored the claim that comics benefit palliative care practice, through evaluating a graphic novel’s value as an aid in supportive conversations with family caregivers.

**Aim:**

To identify facilitators and barriers in using *Naasten* (Loved ones), a Dutch research-based graphic novel about family caregivers providing care at the end-of-life.

**Design:**

Qualitative study, following thematic content analysis.

****Setting/**participants:**

Three focus groups with family caregiver consultants, palliative care volunteers, and healthcare professionals (total *N* = 23) who supported family caregivers; and individual telephone interviews with family caregivers to whom the book was presented (*N* = 4).

**Results:**

Barriers and facilitators related to: (1) the family caregiver, (2) impact on the family caregiver, (3) impact on the conversation between the person who provides support and the family caregiver, (4) their relationship, and (5) the person who provides support. *Naasten* was reported as recognizable and supportive, and powerful in raising emotions, awareness and conversation. Barriers concerned the book’s impact due to its style and guidance of a conversation, and doubts about its surplus-value.

**Conclusions:**

Emotionally impactful comics may support bereaved family caregivers, but should be introduced with care among current family caregivers, for example, ensuring a right fit, introduction, and follow-up—while taking into account a caregiver’s individual situation, needs, abilities, and affinity with the medium. Comics are preferably used in educational settings, contributing to professional awareness and tailored support of family caregivers.

**What is already known about the topic?**

- Family caregivers are highly important in the home setting, but may feel the need to be more visible to healthcare professionals and share their experiences
- To provide tailored support, healthcare professionals and volunteers should be aware of the sometimes implicit perspectives and needs of family caregivers
- The medium of comics has potential for palliative care professionals in raising awareness for people’s personal experiences

**What this paper adds?**

- Participants were ambivalent about the value of our graphic novel as an aid to start a dialog with family caregivers about their experiences: the book raised emotions, recognition and conversations, but was also considered too directive, superfluous or even potentially harmful in support of current family caregivers
- Comics can serve as a window into the lives of others, helping readers to understand and reflect upon the dilemmas faced by family caregivers
- Participants emphasized comics’ educational value for professionals and volunteers in palliative care

**Implications for practice, theory, or policy**

- Comics should be introduced with care in conversations with current family caregivers, due to its possible emotional impact and its effect on the conversation between family caregiver and the person who provides support
- Comics may have educational value in raising awareness of the dilemmas of family caregivers, thus enabling people who provide support to assess a family caregiver’s needs with more specific questions and thus provide tailored support
- Future research should study comics’ value in support practice and in (professional, volunteer, or public) education

## Background

In the last phase of a life-limiting disease, especially at home, the role of a patient’s close ones is pivotal^1^ and intensified.^2^ Partners or family members often find themselves in the role of family caregiver because they are related to the patient.^3,4^ Many family caregivers (hereafter: caregivers), however, live in permanent uncertainty about the future, while feeling unprepared for their caring role^5–7^ and overwhelmed by the demands of the all-consuming nature of caring.^8^ Previous research showed how caregiving impacts normal daily life and social engagements,^2,7,9^ causing physical, emotional, and psychosocial challenges that demand support.^10–13^

Professional care for the patient at home can provide relief^14^ but only if adjusted to the caregivers’ goals and needs in caring.^15^ Caregivers often feel the need to be more visible to healthcare professionals and wish to be considered important members of the caring team.^16,17^ Meanwhile, the phenomenon of family caregiving seems complex and subject to implicit social expectations.^18^ To make healthcare professionals more aware of caregivers’ needs and concerns and to provide tailored support, we developed the Dutch graphic novel *Naasten* (English: Loved ones) which visualizes family care at home for someone with cancer or COPD (Table 1). It has been argued that these graphic stories have potential for palliative care professionals,^19–21^ in obtaining patients’ and families’ personal experiences with serious illness and healthcare.

**Table 1.** About the graphic novel “Naasten.”

**Research-based graphic novel** The 230-page Dutch graphic novel *Naasten* (English: Loved ones) was developed by an interdisciplinary team of researchers and two comic artists who were final-year Comic Design students of ArtEZ, a renowned university of the arts in the Netherlands. The graphic novel tells the stories of characters caring for their loved one receiving palliative care at home, based on themes and scenes from our qualitative interview study with 28 family caregivers (mostly partners or adult children) and 9 patients (mostly suffering from end-stage cancer or severe organ failure).^22^ The larger project, of which this study is a part, aimed to visualize the sometimes invisible experiences of family caregivers, thus stimulating conversations within support practice and among the wider public. **Two interwoven storylines** To provide a general and rich account of what it can mean to provide family care at home, two storylines with different palliative care trajectories were scripted: Geert, who cares for his wife with end-stage cancer; and Eva, who cares for her father with severe COPD. Both family caregivers feel called to care (“this is the last thing I can do”) while balancing it with work, their own needs, changes in the relationship, involvement of friends and other family members, and professionals entering normal life. Each comic artist drew one storyline, in his/her artistic style; the stories are interwoven in the novel. The stories were developed and written by MH and the two art students, under supervision of the students’ art professors who themselves were experienced comic artists. The art students were trained in writing fiction. Please see Supplemental File 1 for examples of the novel’s pages. **Feedback and publication** Both content and form of the graphic novel were critically assessed, from early sketches on throughout the development process. Interviewees (bereaved family caregivers of seriously ill people), palliative care professionals, and other professionals within our project sounding board gave feedback during the development process, mainly with regard to the novel’s recognizability, realism, and tone. The development of the novel was also artistically assessed by the editorial team that included an experienced comics editor and a graphic designer, and by the art students’ art professors. The graphic novel was published commercially by the Belgian-Flemish publisher Oogachtend in 2019. Free copies are available for educational and support purposes (while supplies last).

Graphic memoirs on comic artists’ own illnesses or caregiving experiences have already been used for promoting awareness, for example about mental illness, cancer, dementia, or hospice care.^20,23,24^ It has been argued that the combination of words and images allows comics to *show* how it feels to be ill or to provide care.^25,26^ This showing—sometimes in just one panel instead of large amounts of text—would enable readers to understand the various layers of often complex experiences^21,25–29^: via visual metaphors^30^ or ambiguities^20^ containing multiple messages instead of one absolute meaning.^29^ By creating engagement and affect,^20,21,31^ authors argue, comics facilitate understanding the fictional characters’ perspective.^29,32,33^ Thus, comics offer a window into the subjective lives of others,^21,26^ which enables readers to understand the inner, intangible aspects of illness experiences^34^ and the issues or worries that are not always elicited in clinical encounters.^20,21^

Contrasting autobiographic comics,^28^ our graphic novel (Table 1) was based on our in-depth interview research,^22^ taking up insights from comics-based research.^31^ In general, arts-based research (ABR) practices emerged in the last decades as interdisciplinary and rather innovative ways of conducting, analyzing, or representing research through the arts, for example, poetry, music, dance, theater, or visual arts.^35–37^ Despite the challenges, art-based methods are proposed as worthwhile in qualitative research endeavors and the field of knowledge translation, to understand and communicate complex experiences.^35,37–39^ The potential value of introducing comics in palliative care, specifically, has already been argued,^19^ but empirical research regarding the use and (emotional) impact of comics in healthcare is lacking,^20,24,33,40^ palliative care included.

This study aims to identify facilitators and barriers in the use of comics by palliative care professionals or volunteers as an aid in their supportive conversations with caregivers, thus adding to the evidence base for the value of comics in a palliative care context.

## Methods

### Design and research question

This qualitative study uses thematic content analysis to explore: what are the experienced barriers and facilitators of using the graphic novel *Naasten* as aid in supportive conversations with family caregivers of patients receiving palliative care in the Netherlands?

### Setting, recruitment, and participants

**Figure 1.**
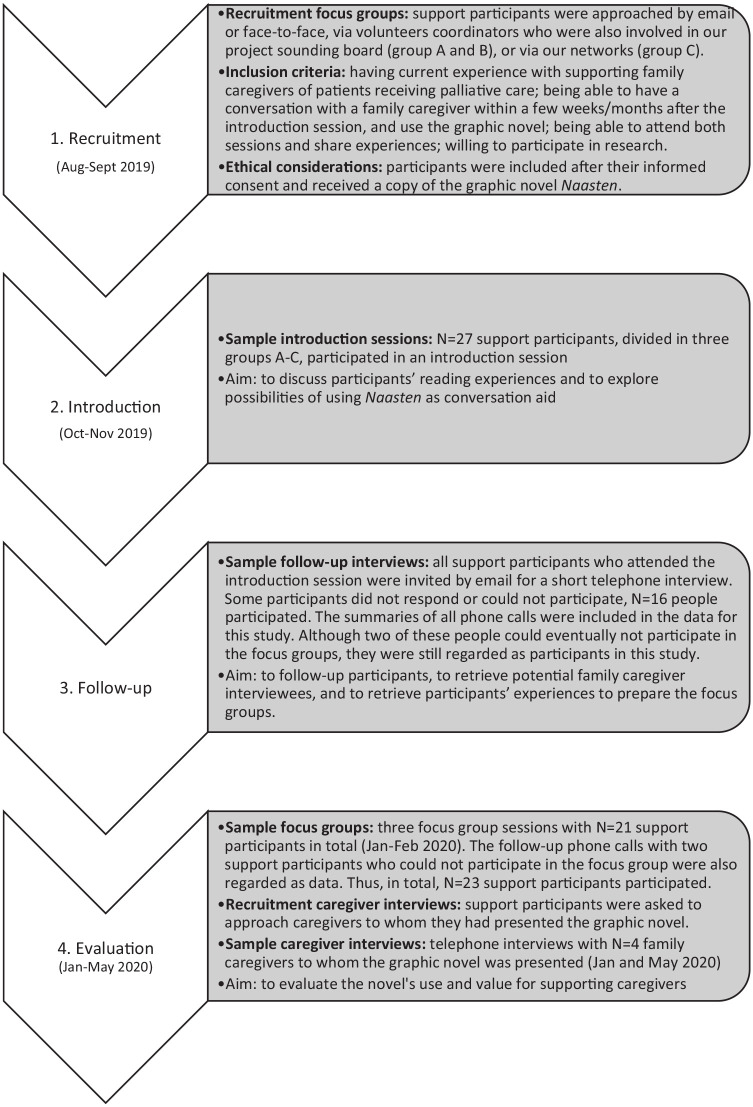
Overview of the steps in the recruitment, sampling, and data collection.

Figure 1 provides details of the recruitment and sampling of participants. To cover diverse views and experiences, and account for triangulation, we included both people who provide support to caregivers (hereafter: support participants) and caregivers:

- Following convenience sampling, we included family caregiver consultants, unpaid palliative care volunteers, and various palliative care professionals in three mixed groups (Figure 1, step 1). Volunteers were recruited via volunteers coordinators. All participants received the graphic novel in an introductory training setting.
- These support participants were asked to approach caregivers to whom they had presented the graphic novel, thus leading to inclusion of interested caregiver participants via convenience sampling (Figure 1, step 4).

### Data collection

- The introduction sessions with 27 participants (Figure 1, step 2) were guided by GO, an associate professor with ample experience in qualitative research, assisted by the volunteers’ coordinator involved in our project and experienced research-assistant MK. These sessions were not audio-recorded or analyzed because of their introductory and training-nature.
- Follow-up telephone interviews with 16 support participants (Figure 1, step 3) were conducted, audio-recorded and summarized by MK.
- We wanted to enable participants to learn from their fellow participants while sharing and discussing their experiences with using *Naasten* in their (volunteering) work. Therefore, we conducted focus groups with the same groups of participants as the introductory sessions, assuming that their interaction would help to explore and clarify the participants’ possibly diverse opinions.^41^ Three focus groups with 22 participants (Figure 1, step 4), each with between six and eight support participants, lasted 88 min on average (range 77–94, 5 min). These were moderated by GO, using a semi-structured topic guide with open-ended questions (Supplemental File 2) and assisted by the volunteers’ coordinator who took field notes. MH also assisted groups B and C.
- Four individual telephone interviews with caregivers (Figure 1, step 4) were conducted by MK, guided by a semi-structured topic guide with open-ended questions (Supplemental File 3) and lasted 32 min on average (range 22–55, 5 min). Telephone interviews were considered to be least invasive.

### Data analysis

All focus groups and caregiver interviews were audio-recorded and transcribed verbatim. The transcripts were coded, assisted by ATLAS.ti 8.3 software, following five steps of thematic content analysis.^42^ The eventual categories and domains were determined in an iterative process during initial coding, categorizing, and writing of the manuscript; there was no a priori coding framework (Supplemental File 5). To ensure familiarization and generation of initial codes (step 1–2), one focus group transcript and one interview transcript were independently coded by MK and MH. Discrepancies were discussed until consensus was reached and a first codebook with categories (step 3–4) was developed. MK then coded all other transcripts, with MH intermediately checking the codes and making remarks. While producing the manuscript (step 5), and supported by project team review, MK tightened the codebook and defined the categories and larger domains (step 4). Because of this study’s explorative nature, data saturation was not expected.

### Ethical considerations

Ethical approval was sought from the Research Ethics Committee of the Radboud University Nijmegen Medical Center (registration number 2017-3415), who determined ethical approval was not required under Dutch law. All participants gave their written consent, after receiving an information flyer. Pseudonymized data were safely stored, as was stated in an approved data management plan. The research was reported following the Consolidated Criteria for Reporting Qualitative Research (COREQ) guideline^43^ (Supplemental File 4).

## Results

In total, 23 support participants (22 female, 1 male) and 4 family caregivers (3 female, 1 male) were included (Figure 1, step 3 and 4; Tables 2 and 3). Six support participants dropped-out after the introduction sessions (three of group B, three of group C) due to personal circumstances or lack of time; two of them still participated in the follow-up telephone interviews.

**Table 2.** Support participant characteristics.

Method	Group	Participants									
		Volunteer	Family caregiver consultant	Social worker in training	Nurse (in palliative or hospice care)	General practitioner (in training)	Nursing home physician	Spiritual counselor (in training)	Hospice employee family care	Psychologist	Total *N*
Introduction session (Figure 1, step 2)	A	4	3		1						8
	B	6	2	1							9
	C				2	2	1	3	1	1	10
											27
Follow-up telephone interviews (Figure 1, step 3)		6	5	1		2		2			16
Focus groups (Figure 1, step 4)	A	4	3		1						8
	B	3	2	1							6
	C				1	2	1	3			7
											21

**Table 3.** Family caregiver participant characteristics.

	Gender	Age (mean 65.75)
1	Female	78
2	Female	59
3	Female	63
4	Male	63

Overall, the support participants reported diverse ways of using the novel in their daily practices: they browsed through *Naasten* together with caregivers, focused on specific images or storylines, used separate images as association cards, had conversations after the caregivers had read the novel themselves, placed it in a public place (e.g. waiting room, living room or library), or showed it just to colleagues/acquaintances and not to caregivers. Several participants did not use the novel at all. These diverse ways were based on the supporter’s considerations about, for example, one’s personal opinion about the book, time, the relationship with the caregiver, or the character of the conversation. Some participants reported not having caregivers to whom the book was fitting.

On the one hand, the graphic novel *Naasten* (Table 1; Supplemental file 1) was praised for its originality, recognizability, ability to touch readers, and the portrayal of moments of tender loving care. On the other hand, it was disregarded as being too dark and sad, confronting, complicated due to the two interwoven and rather differently drawn storylines, or just “too much” being a 230-page book.

Based on the diverse experiences, we identified: A) facilitators and barriers in using *Naasten* as conversation aid (Table 4), and B) the novel’s potential value in education.

**Table 4.** Overview of facilitators and barriers in using “Naasten” as conversation aid.

Concerning these domains: ↓	Facilitators	Barriers
1. The family caregiver	Comics in general being easily accessible	Misfit with the specific person, phase, or setting
2. Impact on the family caregiver	Being recognizable and supportive, and raising awareness about family care	Being too confronting and not supportive in conversations
3. Impact on the conversation between the person who provides support and the family caregiver	- Raising specific conversation topics, deepening the conversation - Raising awareness among people who provide support in palliative care, evoking specific questions	Being too directive and having no surplus-value with regard to topics being discussed
4. Relationship between the person who provides support and the family caregiver	Existing relationship with the family caregiver, possibility of follow-up	Short-time contact, risk of damaging relationship or image as a professional
5. Person who provides support to family caregivers within palliative care	Enthusiasm about, access to, and familiarity with *Naasten*	Unfamiliarity with the comics medium

### Facilitators and barriers in using Naasten as aid in supportive conversations with caregivers

#### Domain: The family caregiver

##### Facilitators: Comics being easily accessible

Participants looked positively at comics’ focus on *showing* caring experiences without using much text. This made the graphic novel easily accessible to caregivers, for example people with language difficulties or a migrant background. It was also suggested that comics could suit caregivers who cannot concentrate on reading texts:

*Well, I think that is a very good idea because you know, when you are in misery, because you are, you have no. . . You do not feel like reading. If you read two sentences you do not remember what was written before that. You have no room in your head. And pictures are just easier. So yes, I think it’s a really good idea. Because you just. . .you just can’t read. You can’t remember it, you can’t concentrate at this time, especially when you are feeling that wretched. (family caregiver 2)*

Another suggestion was to use the images as a conversation starter for people who find it difficult to express what is on their minds.

##### Barriers: Misfit with the specific person, phase, or setting

A misfit between the novel’s setting and the caregiver’s own story and experiences was considered as hindering. Affinity with visual language was also important.

Some support participants were interested in using separate images, others reported their impression that separate images or graphic metaphors were not always understood by caregivers:

*That gentleman with COPD. Then you see that entire mountain, you know. And someone asked, “Is he still going to Austria, even though he is that sick?” I said, “No. For him it’s already a. . . He’s so short of breath, so short of breath. That threshold is already too much for him. Especially if he dreads it that much.” (in response to a page of the book) (participant of focus group B) (see Supplemental File 1, image 2)*

#### Domain: Impact on the family caregiver

##### Facilitators: The novel being recognizable and supportive, and raising awareness

Some participants found *Naasten* less representative for the loving moments in care, others thought it rightly depicted the “*horrible time*” of caring in the last phase. In general, the novel was considered recognizable by both support participants and caregivers. This was supportive to some:

*“Deeply impressed with the way feelings are expressed here. I myself had to leave my mother behind after an illness. (. . .) The illustrations recreate this feeling. It is somehow good to know that this feeling is also experienced by others. Recognition. Very beautiful and valuable.” (during the course of focus group B, a participant reads out loud what an acquaintance wrote to her about the book)*

It was both hypothetically argued as well as reported that this recognizability can stimulate caregivers to ask for help in time when feeling overburdened. The novel was considered helpful for caregivers to reflect and raise awareness of their needs:

Participant X: *Sometimes family care sneaks up on you and is a matter of course. And it is almost an obligation because it comes about naturally. And the person involved does not see it as taxing at all. But the outside world does see it like, “Hey. . .” Then you could also say, “Well, look, this is how others deal with it, or you can solve it like this. You don’t have to do it all by yourself.” And then a book like this can also. . .*

Participant Y: *. . . Hold up a mirror. Make you reflect.*

*(participants of focus group A)*

##### Barriers: The novel being too confronting and not supportive

A downside of the novel’s recognizability was that its display of deterioration and death was reported as potentially too confronting for current caregivers, especially when already overburdened. Some caregivers reported feeling miserable after reading, or even being appalled by the black-and-whiteness of one storyline:

*No, if it should be a book to support me, then I think, never mind, because it made me very sad and glum. Especially because of the drawings made by one of the two. There are two illustrators and one has a very. . . The drawings are sketched very harshly and are dark and depressing. Without really looking at the details of the drawing, but just by looking at the paper it was drawn on, it gave me a very gloomy feeling. (. . .) I can’t say that I remember anything. Not the recognition or the support it is meant to give, it rather made me feel down. I think that’s sad. (family caregiver 3) (Supplemental File 1, image 3)*

Due to the evoked emotions, support participants reported being hesitant in using *Naasten* as an aid in their conversations. We observed a tendency among study participants to protect caregivers, fearing to confront them and worsen their situation:

*Yes, I also thought a little like, “hey, isn’t it too confrontational? Am I not touching on too much?” Because I also thought that the darkness, that it is very confronting. And I’m already touching on a period of major grief, so am I not making it even worse? But then I also think that sometimes it can be a good thing that you might make matters even worse at that moment. And we are often a little hesitant about that, but that is when you can dig deeper. (participant of focus group A)*

Participants argued that *Naasten* would better suit bereavement support.

#### Domain: Impact on the conversation between the person who provides support and the caregiver

##### Facilitator: Raising specific conversation topics, deepening the conversation

Using separate images as associative cards in a caregiver support group was found to stimulate a conversation about each other’s experiences. According to a volunteer, a young caregiver criticized the novel’s clichés but also elaborated on how her experiences were different than the character’s. Another volunteer explicitly reported having in-depth conversations due to certain images:

*Well, for example, at one point there was a very profound conversation with the young caregiver about the question of guilt. I don’t know that if I hadn’t had a picture of that daughter with her father, whether you could have dug that deep. (. . ..) Like that young caregiver, she clearly looked at the dark images of the daughter with the father. And that was her situation, and so at one point she got around to well, this story, “I have to go there because . . .” (. . .) Well, and so the story was told, “I should have stayed with you on the last day. I left in the morning, and my father was not well. And I did pass by the general practitioner, but he actually really didn’t want me to, and my father said I had to go to school. So, I did go to school. And then you come back in the afternoon and he’s lying at the bottom of the stairs.” That’s when the feeling of guilt occurred, but it was only because of this picture, then it came up (in response to a page of the book). (participant of focus group A, volunteer) (Supplemental File 1, image 4)*

##### Facilitator: Raising awareness for supportive people, and thus evoking specific questions

Most participants were ambivalent about the novel’s value as an aid in supportive conversations. However, they reported its potential for raising (renewed) awareness of caregiver experiences and needs among professionals, volunteers, or family members surrounding a caregiver. Certain experiences for which one might have a blind spot might be addressed in a conversation with the caregiver when thought necessary or helpful:

*When we have a conversation, it may well be that we also have a blind spot or that it does not come up spontaneously. (. . .) But this [book, MH] is based on the interviews you’ve done. So, those are subjects or topics that people have mentioned. I don’t know whether I, as a care provider, would spontaneously bring up all these different subjects or spot them easily. I can imagine that there are topics in the book that otherwise would not surface. Something someone is still struggling with, for example. (participant of focus group C) (Supplemental File 1, image 5)*

##### Barriers: Too directive and having no surplus-value

Using *Naasten* instrumentally as a conversation aid felt unnatural considering the risk of steering the conversation with a caregiver too much. According to both support and caregiver participants, people should *listen* first to what is on another one’s mind, instead of making interpretations about the other’s feelings, or starting a conversation with prescribed themes:

*Well, what my neighbour [fellow focus group participant, MH] said about ‘unnatural’ I sometimes felt that too. In the sense of. . .well, my first inclination when I’m talking to someone is to just listen and see what comes up. What is going on with people, to be very open and to not directly offer something. I can imagine that if you are in this situation, that when people themselves might come out with some of the dilemmas that play out in that book, that you then grab that book. I haven’t had that happen lately. (participant of focus group C)*

Some reported not needing the book to discuss certain themes.

#### Domain: Relationship between the person who provides support and the caregiver

##### Facilitators: Existing relationship with the family caregiver, possibility of follow up

Support participants emphasized the importance of having an existing relationship with the caregiver, enabling them to “sense” if *Naasten* would fit the setting and a caregiver’s current situation.

*I also recognize the hesitation mentioned before, like, do you start out with your own approach or have you come to listen to someone? Only this lady started talking about her sister who had just lost her husband to cancer. She came from [a country abroad, MH] and so her sister was also in [country abroad, MH]. So, she was a relative at distance from her sister. I said, “I have a book in my bag, in which both stories, your story and your sister’s story, partly come together and run side by side.” So, then we got into a conversation about this book. So that was great. (participant of focus group C)*

It was also recommended to take enough time for the conversation, and to read the novel together instead of handing it out to caregivers—unless one would assess the caregiver being capable of handling the possible emotional consequences. Nonetheless, follow-up care should be guaranteed:

*It also seems to me that that is actually a requirement to give the book, well, to people, to talk to people about it. Anyway, at the very least you should say, if you want to talk about it, I’m always there for you or something along those lines. (Interviewer: Yes, so the possibility to talk about the book after it has been read should always be there.) Yes, and then also about yourself, because that is, ultimately, what it is all about. That you recognize yourself in it and can do something with it. And in order to achieve that, I don’t think you should hand out this book just like that. (family caregiver 1)*

##### Barriers: Short-time contact, risk of damaging relationship or image

A conversation about the novel was regarded as “going deep” and therefore unsuited for first, one-time, or brief contacts, without follow-up:

*I just have short-term contact with people. The moment - I can’t give this book to someone I’m just getting to know. I am a caregiver consultant. (. . .) Sometimes you dig down deep, but then this is not the first thing you show them, not to someone you don’t know. (. . .) Because at first you have to give them the space to tell their own story. So, you have to get to know them. This goes in-depth immediately. I want to be a little careful with that when it comes to the initial conversation. (. . .) The way I see it is that you simply can’t do everything in one conversation. I want to protect people from that. People are already in over their heads. They have to tell their own story first. Do you also want to immediately share with them the deeper layer, revealed by the topics of the stories in this book? I actually don’t agree with that. (participant of focus group B)*

Some feared using the book would trouble or even damage their relationship with the caregiver, or their image as a professional.

#### Domain: The person who provides support to caregivers within palliative care

##### Facilitators: Enthusiasm about, access to, and familiarity with the book

Facilitating for integrating *Naasten* in supportive conversations was participants’ own enthusiasm, curiosity regarding its possible value, familiarity with its themes, and always carrying the novel with them. Experience with showing the book to caregivers made it easier to suggest it to others:

*It is essentially about giving and about doing. And if you also have it [the book, MH] with you and you think at that moment. . . I always carry something with me, like cards for example, but I could also use this book. (. . .) Yes, it has become a little easier to show it because you’ve used it more often now. (participant of focus group A)*

##### Barriers: Unfamiliarity with the medium

Some support participants questioned the novel’s surplus-value in comparison to other methods or their professional skills. A barrier was that the comics medium was relatively new. Some felt hesitant in using the novel as opposed to trusting their own (more guidable) conversation techniques:

Participant X: *Well, maybe there is also this fear that is not really necessary. Of course you know about your own . . . You can rely more on your own conversational technique or communication and you know you can guide it more easily than sharing something that might . . .*

Participant Y: *That it doesn’t work at all, for instance. That he thinks: “What are you showing me this time?”*

Participant X: *Yes, exactly! And that you think, “Oh, yes. Well.” And then you can imagine that it will be uncomfortable – so to speak*.

*(participants of focus group C)*

### The potential value of the graphic novel in education

In all focus groups, participants considered *Naasten* to be informative for a variety of people providing home-based palliative care. Its potential value as an educational tool for professionals or volunteers in training was also emphasized by caregivers themselves, in raising awareness concerning their presence and needs:

*“I speak from experience and even got very emotional when reading the book, it immediately brought me back to that ‘hopeless’ and extremely busy period when nothing else existed but caring for and organizing. I felt myself once again being pulled apart by my company, my family and my father who needed so much attention and care. (. . .) I advise anyone who will have to deal with family care in the future (and that includes all of us in the Netherlands) to read this book to be better prepared for what is to come. It would therefore be great if this is already available at schools and during training.” (Participant of focus group B reads out loud during telephone interview what an acquainted family caregiver wrote to her)*

Consequently, it was argued, *Naasten* might enable students, for example in nursing, to ask more specific questions and provide better support:

*The book can help people in training to see which topics are at play and once the professionals start working with family caregivers, he or she can draw on that and think, hey, that subject was mentioned in the book. I have to dig a little deeper. What specific questions can I ask to help this caregiver? (family caregiver 4)*

## Discussion

### Main findings

Based on a qualitative analysis of facilitators and barriers in using the Dutch graphic novel *Naasten* about family care at home (Table 1, Supplemental File 1), this study offers some support for the claim that comics can benefit palliative care practice. Participants were ambivalent about its use as an conversation aid: *Naasten* was recognizable, raised emotions, awareness, and (in-depth) conversations, and may support bereaved caregivers; it was also reported to be potentially harmful due to its emotional impact, too directive in the conversation, or superfluous. Comic art should thus be introduced with care among caregivers. Preferably, comics are used in educational settings, contributing to professional awareness and tailored support of family caregivers.

### What this study adds

With respect to comics’ potential for palliative care^19,21^ and specifically for supporting family caregivers, our results suggest that, first, *Naasten* indeed served as a window into the lives of others,^21,26^ raising issues or conversations that probably would not have taken place otherwise. Comics may help caregivers reflect on often-implicit norms and values, which is deemed important^44^ as family caregiving is shown to be subject to social expectations.^18^

Secondly, comics should be introduced with care. Comics’ direct way of showing experiences,^25,26^ without apology,^45^ evoked contrasting responses. *Naasten* was found original and recognizable, and therefore supportive. Participants, however, also considered the novel too directive, simply superfluous, or even potentially harmful due to its emotional topics. A barrier for its use in supportive conversations was the impression that *Naasten* was too confronting for sometimes already overburdened caregivers. Previous research also reported this critique on distressing and pessimistic health comics.^29^ An important question for support in palliative care is whether confrontational comics actually “*enhance (rather than dismantle) existing coping strategies*” (p. 7),^46^ thus contributing to the quality of life of patients and families.^47^

Third, comics’ seeming accessibility can be questioned. Participants suggested that the medium may suit overburdened caregivers unable to concentrate on much text. Some caregivers, however, showed difficulties with understanding visual metaphors,^30^ contesting the argument that the medium can communicate complex things in a concise way.^27^ Comics demand active interpretation of the narrative and everything that is *implied* ^20,26,29,45^—having to slow down to fully grasp a comic^48^ may not fit a turbulent palliative care context.

Overall, our results indicate that *Naasten* raises emotions and conversations, and may support bereaved caregivers. We should, however, not overestimate the possibilities of using emotionally impactful comics in supportive conversations with people who currently provide family care. Their value in education may be greater. It has already been argued that art can be powerful in educating healthcare professionals.^38,49–51^ Comics, specifically, may create awareness and understanding through using characters and narrative.^29^ There may also be more time in educational settings, compared to palliative care practice, to reflect on comics and their (implicit) meanings. We hypothesize that by gaining a better understanding of caregivers’ perspective through reading comics such as *Naasten* in an educational setting, people who provide support are enabled to assess an caregiver’s perspective and needs and with more specific questions. Thus, we argue, reading comics may contribute to better supportive care and to the reported need of caregivers to being more visible to healthcare professionals.^16,17^

### Future research

The results of this explorative study provide sufficient reason to further investigate comics’ application and value, mainly in palliative care education. Our participants were experienced professionals and volunteers who recognized the graphic novel’s scenes and themes in their daily practices. Future research should be focused on students or volunteers in training,^19^ for example, fostering sensitivity for the perspective of partners and families within palliative care. Another area is public education and awareness, as most investigated palliative care education programs are targeted at healthcare professionals and caregivers.^52^

### Implications for practice

Emotionally impactful comics, such as *Naasten*, may support bereaved caregivers. When considering using comics in conversations with current caregivers, we would advise to assure:

- a right fit between the comic’s content and the caregiver’s situation, setting, and phase, which requires a time investment and sensitivity to both the comic’s content and the individual’s situation^53^
- a caregiver’s affinity with the visual medium
- a caregiver’s ability to view or read possibly confrontational images
- a careful introduction and follow-up, possibly within a trusting (long-term) relationship
- a conversation directed by what prompts the caregiver to react

Furthermore, we have experienced that stimulating conversations among professionals and volunteers works best when discussing pages from the novel in-depth:

(1) What do you see?
(2) What do these panels evoke?
(3) What does this tell you about family caregiving, and about caregivers’ moral dilemmas and values?
(4) What do you gain from this for your daily practice?

### Strengths and limitations

To our knowledge, this study’s strength is that it is the first to empirically explore the use of comics within the context of palliative care. A limitation was this study’s explorative nature and the heterogeneous use of our novel. This problematized its generalizability, similar to previously described difficulties with measuring “impact” in an interdisciplinary art-science project^54^ and consistently replicating artistic interventions.^55^ Another limitation was the relative brief window of opportunity in palliative care settings for implementing new support methods,^46^ such as our novel, hindering a precise sampling of participants and making us dependent on convenient sampling. Lastly, the volunteer coordinators involved in our research project recruited participants they already knew. This hindered a systematic collection of sociodemographic variables and may have caused a bias.

### Concluding remarks

Through *showing* (instead of telling about) lived experiences of family caregivers, our graphic novel can be used as an aid in supportive conversations, to raise certain topics with family caregivers. Such emotionally impactful comics, however, should be introduced with care, for example, ensuring a right fit, introduction, and follow-up—while taking into account the caregiver’s individual situation, needs, abilities, and affinity with the medium. Comics are preferably used in educational settings to stimulate a dialogue about family caregiving among healthcare professionals and volunteers, contributing to their awareness and tailored support of family caregivers.

#### Supplemental Material

sj-pdf-1-pmj-10.1177_02692163221093513 – Supplemental material for Facilitators and barriers in using comics to support family caregivers of patients receiving palliative care at home: A qualitative study Click here for additional data file. Supplemental material, sj-pdf-1-pmj-10.1177_02692163221093513 for Facilitators and barriers in using comics to support family caregivers of patients receiving palliative care at home: A qualitative study by Maaike M Haan, Jelle LP van Gurp, Marjan Knippenberg and Gert Olthuis in Palliative Medicine

sj-pdf-2-pmj-10.1177_02692163221093513 – Supplemental material for Facilitators and barriers in using comics to support family caregivers of patients receiving palliative care at home: A qualitative study Click here for additional data file. Supplemental material, sj-pdf-2-pmj-10.1177_02692163221093513 for Facilitators and barriers in using comics to support family caregivers of patients receiving palliative care at home: A qualitative study by Maaike M Haan, Jelle LP van Gurp, Marjan Knippenberg and Gert Olthuis in Palliative Medicine

sj-pdf-3-pmj-10.1177_02692163221093513 – Supplemental material for Facilitators and barriers in using comics to support family caregivers of patients receiving palliative care at home: A qualitative study Click here for additional data file. Supplemental material, sj-pdf-3-pmj-10.1177_02692163221093513 for Facilitators and barriers in using comics to support family caregivers of patients receiving palliative care at home: A qualitative study by Maaike M Haan, Jelle LP van Gurp, Marjan Knippenberg and Gert Olthuis in Palliative Medicine

sj-pdf-4-pmj-10.1177_02692163221093513 – Supplemental material for Facilitators and barriers in using comics to support family caregivers of patients receiving palliative care at home: A qualitative study Click here for additional data file. Supplemental material, sj-pdf-4-pmj-10.1177_02692163221093513 for Facilitators and barriers in using comics to support family caregivers of patients receiving palliative care at home: A qualitative study by Maaike M Haan, Jelle LP van Gurp, Marjan Knippenberg and Gert Olthuis in Palliative Medicine

sj-pdf-5-pmj-10.1177_02692163221093513 – Supplemental material for Facilitators and barriers in using comics to support family caregivers of patients receiving palliative care at home: A qualitative study Click here for additional data file. Supplemental material, sj-pdf-5-pmj-10.1177_02692163221093513 for Facilitators and barriers in using comics to support family caregivers of patients receiving palliative care at home: A qualitative study by Maaike M Haan, Jelle LP van Gurp, Marjan Knippenberg and Gert Olthuis in Palliative Medicine

sj-pdf-6-pmj-10.1177_02692163221093513 – Supplemental material for Facilitators and barriers in using comics to support family caregivers of patients receiving palliative care at home: A qualitative study Click here for additional data file. Supplemental material, sj-pdf-6-pmj-10.1177_02692163221093513 for Facilitators and barriers in using comics to support family caregivers of patients receiving palliative care at home: A qualitative study by Maaike M Haan, Jelle LP van Gurp, Marjan Knippenberg and Gert Olthuis in Palliative Medicine
