# Supplementary material for: Facilitators and barriers in using comics to support family caregivers of patients receiving palliative care at home: A qualitative study
Source: Palliat Med. 2022 May 3;36(6):994–1005. doi: 10.1177/02692163221093513 (PMC9174613; doi:10.1177/02692163221093513)
Supplement: sj-pdf-1-pmj-10.1177_02692163221093513 – Supplemental material for Facilitators and barriers in using comics to support family caregivers of patients receiving palliative care at home: A qualitative study [file sj-pdf-1-pmj-10.1177_02692163221093513.pdf]

Examples of pages from the Dutch graphic novel *Naasten* about family caregiving at home

Information about the book

The 230-page Dutch graphic novel *Naasten* (English: Loved ones) tells the stories of characters caring for their partner or family member receiving palliative care at home. It was based on themes and scenes from the qualitative interview study[1] that was part of our larger research project. To provide a general and rich account of what it can mean to provide family care at home, two storylines with different palliative care trajectories were scripted: Geert, who cares for his wife with end-stage cancer; and Eva, who cares for her father with severe chronic obstructive pulmonary disease. Both family caregivers feel called to care ("this is the last thing I can do") while balancing it with work, their own needs, changes in the relationship, involvement of friends and other family members, and professionals entering normal life. Comic artists Melanie Kranenburg and Niek van Ooijen each drew one storyline, in their own artistic style; the two stories are interwoven in the novel. The graphic novel was published commercially by the Belgian-Flemish publisher Oogachtend in 2019. Free copies are available for educational and support purposes (while supplies last). The art work in this supplemental file was submitted with permission of the comic artists.

Image 1: Cover of the book, showing the title Naasten (Loved ones) and the two main characters

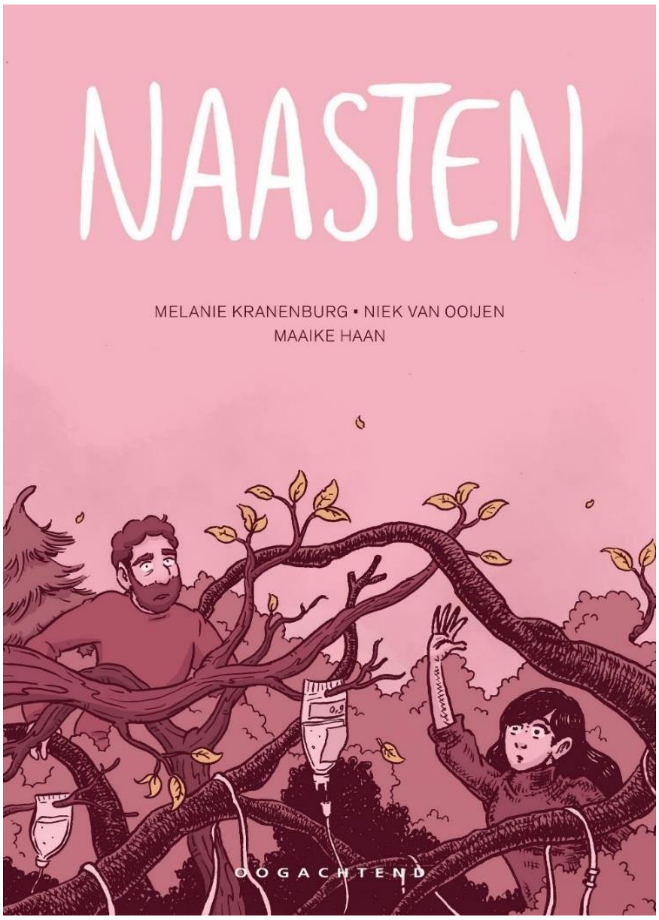

[1] Haan, M.M., Olthuis, G. & van Gurp, J.L.P. Feeling called to care: a qualitative interview study on normativity in family caregivers' experiences in Dutch home settings in a palliative care context. BMC Palliative Care 20, 183 (2021). https://doi.org/10.1186/s12904-021-00868-2

Image 2: Use of visual metaphors in the storyline of daughter Eva and her father with a lung disease

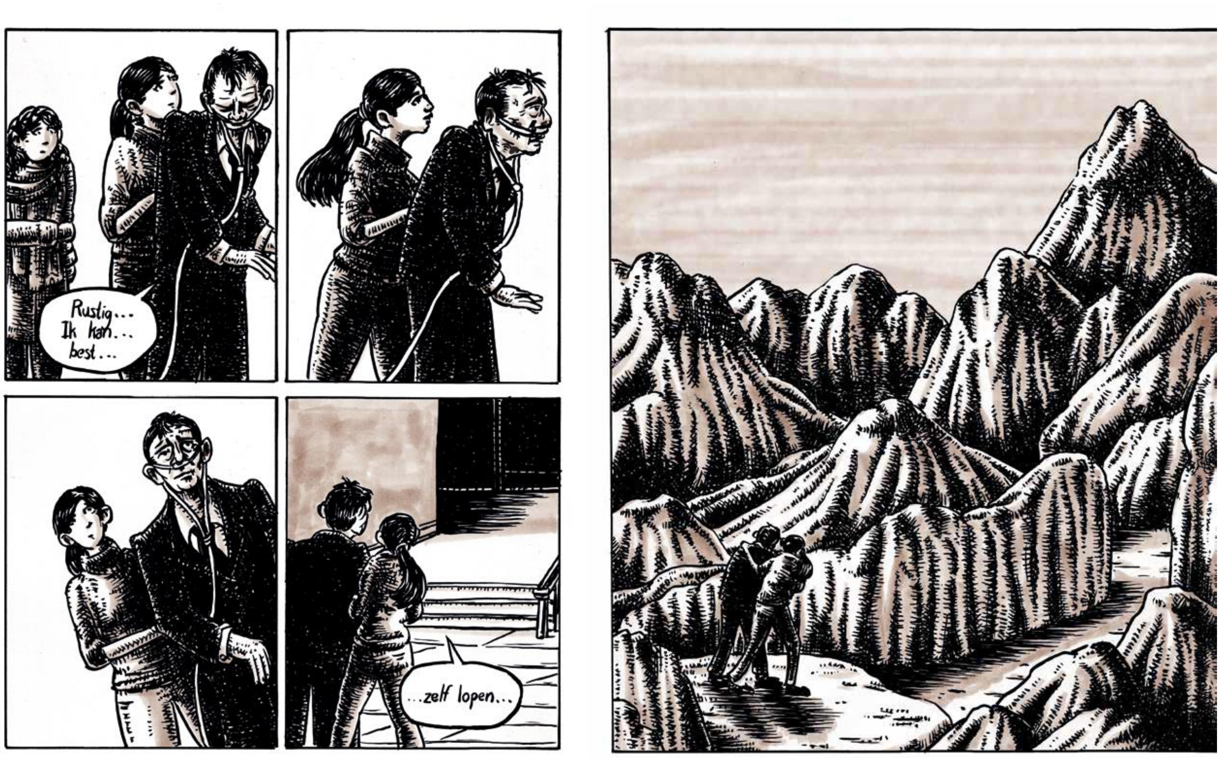

(*Father:* “Easy … Sure … I can … walk by myself …”)

©Kranenburg, Van Ooijen, and Haan (2019), p. 116-117

Image 3: Two storylines drawn in different styles, depicting Geert caring for his wife and daughter Eva caring for her father

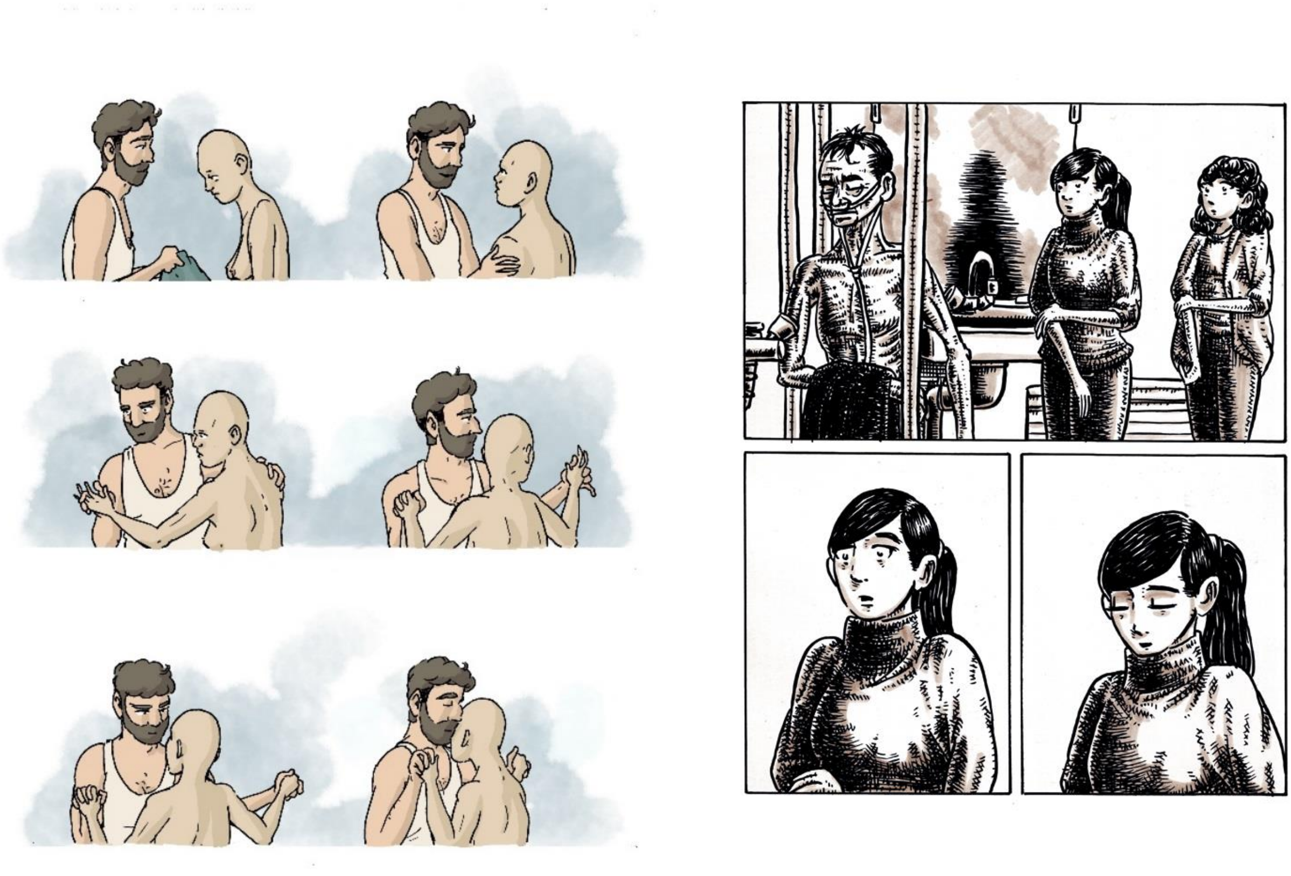

©Kranenburg, Van Ooijen, and Haan (2019), p. 62 and p. 119

Image 4: Daughter Eva responding to an urgent call from her father in the middle of the night, reflecting the pressure in family caregiving in the last phase of life

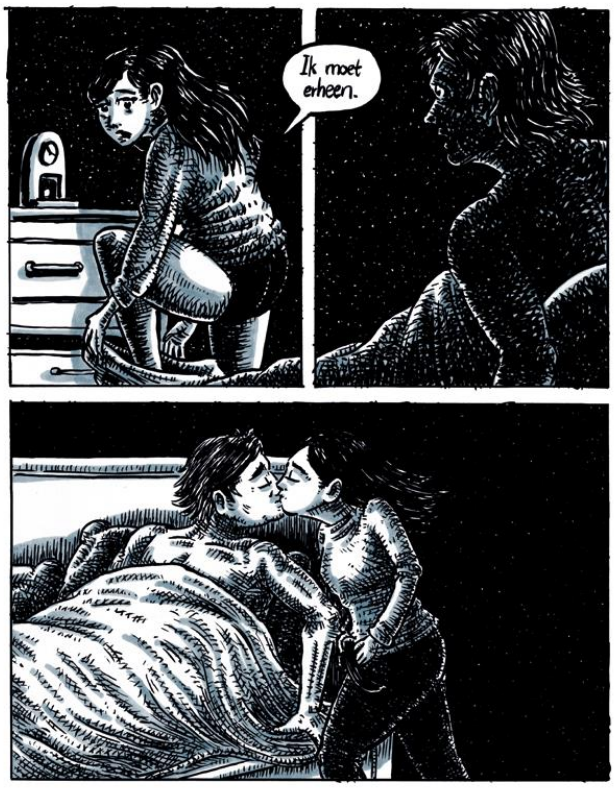

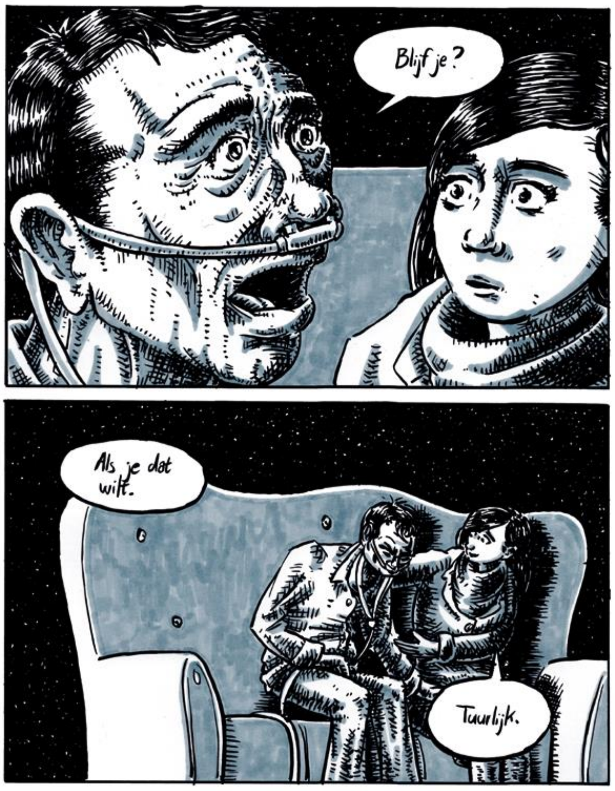

(Eva to her partner: "I have to go there.")

(Father: "Will you stay?"
Eva: "If you want me to. Sure.")

©Kranenburg, Van Ooijen, and Haan (2019), p. 169 and p. 179

Image 5: Example of things a family caregiver may struggle with

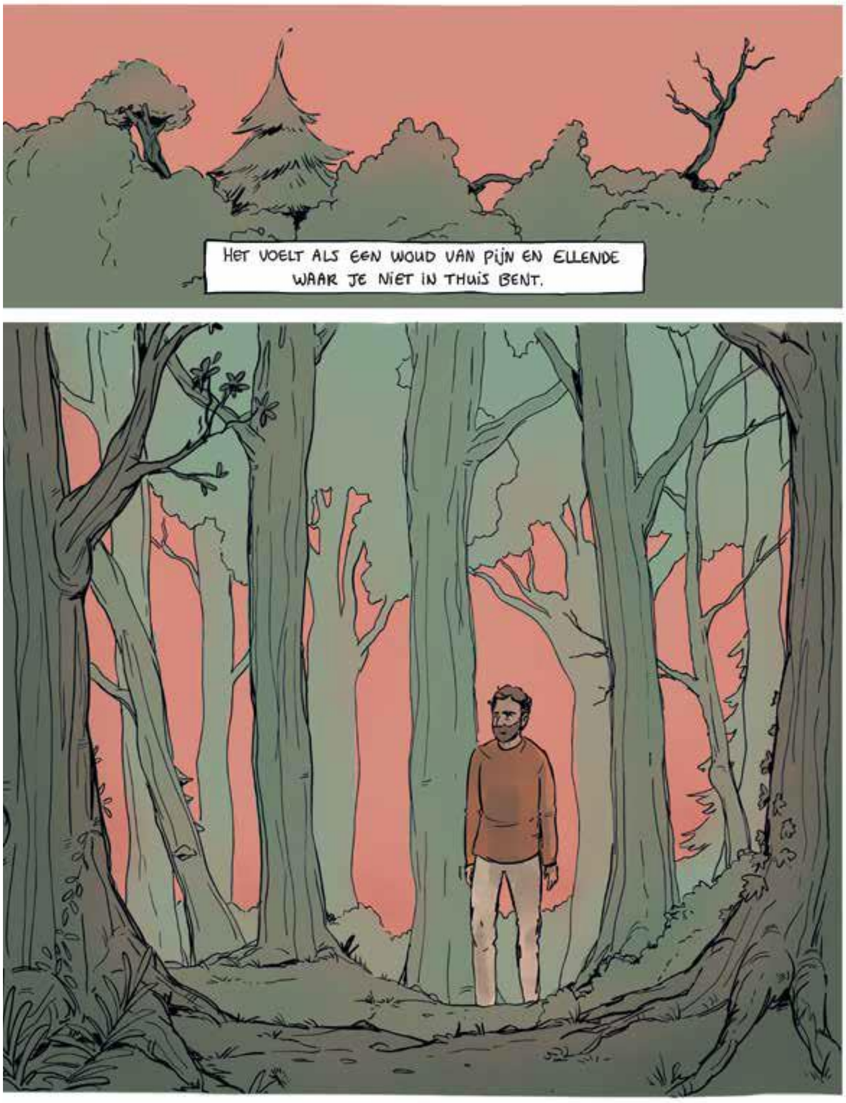

("It is like being in an unfamiliar forest of pain and misery.")

©Kranenburg, Van Ooijen, and Haan (2019), p. 89
