# Supplementary material for: Facilitators and barriers in using comics to support family caregivers of patients receiving palliative care at home: A qualitative study
Source: Palliat Med. 2022 May 3;36(6):994–1005. doi: 10.1177/02692163221093513 (PMC9174613; doi:10.1177/02692163221093513)
Supplement: sj-pdf-2-pmj-10.1177_02692163221093513 – Supplemental material for Facilitators and barriers in using comics to support family caregivers of patients receiving palliative care at home: A qualitative study [file sj-pdf-2-pmj-10.1177_02692163221093513.pdf]

Topic guide for our focus groups with professionals and volunteers who supported family caregivers

- How did you use the graphic novel *Naasten*?
- What were your experiences with presenting *Naasten* to family caregivers?
    - What was facilitating or hindering?
    - How did the people who received the novel respond?
- What differences did you experience between what you expected and what really happened?
- For what type of family caregiver would this book be suited to use? How do you judge to whom the book is suited and to whom not?
- In what way can this book be valuable? Can you give concrete examples where a conversation about a certain topic became easier?
- Do you have recommendations for using the novel?
