# Supplementary material for: Facilitators and barriers in using comics to support family caregivers of patients receiving palliative care at home: A qualitative study
Source: Palliat Med. 2022 May 3;36(6):994–1005. doi: 10.1177/02692163221093513 (PMC9174613; doi:10.1177/02692163221093513)
Supplement: sj-pdf-3-pmj-10.1177_02692163221093513 – Supplemental material for Facilitators and barriers in using comics to support family caregivers of patients receiving palliative care at home: A qualitative study [file sj-pdf-3-pmj-10.1177_02692163221093513.pdf]

Topic guide for our telephone interviews with family caregivers

- Personal situation: what is your relationship with the person you are/were taking care of? How would you describe the caring situation?
- Experiences with the graphic novel Naasten
  - How was the graphic novel *Naasten* presented to you?
  - Did you read the novel? What did you feel about reading it?
  - What did reading the novel evoke?
  - In your opinion, what was positive about the novel? What was less positive?
  - In case of retrospective interviews: do you think your experiences would have been different if you were still taking care of your loved one?
- Experiences with the conversation based on the graphic novel
  - Did you have a conversation with the volunteer/professional? What did you discuss? Did the novel help to begin the conversation?
  - Did the novel had surplus value in the conversation? If yes, in what way?
  - What do you think about the fit of *Naasten* as means to start a conversation or to discuss certain topics?
  - Do you have recommendations for using the novel?
