# Supplementary material for: Facilitators and barriers in using comics to support family caregivers of patients receiving palliative care at home: A qualitative study
Source: Palliat Med. 2022 May 3;36(6):994–1005. doi: 10.1177/02692163221093513 (PMC9174613; doi:10.1177/02692163221093513)
Supplement: sj-pdf-5-pmj-10.1177_02692163221093513 – Supplemental material for Facilitators and barriers in using comics to support family caregivers of patients receiving palliative care at home: A qualitative study [file sj-pdf-5-pmj-10.1177_02692163221093513.pdf]

Supplemental File 5. Coding tree

Explanatory note
The following pages present an overview of the coding tree. In our thematic content analysis following Braun & Clark (2006), we did not use an a priori coding framework. Rather, the codes and categories were determined during an iterative process of initial coding and categorizing. We thereby focused on which factors were positively or negatively contributing to using the graphic novel as conversational aid.

Initially, the codebook consisted of the following categories: Content of the book, Form of the book, Methods of using of the book, Barriers in using the book, Facilitators in using the book, Impact of the book, Needs of the participants, Suggestions of the participants, and Target audiences for the book. When critically re-reading the quotations associated with the codes and categories during our writing down of the results, we renamed or broke down some of the initial codes, specified the categories, and identified the larger domains to point out a pattern with regard to which factors were facilitating or hindering for what and to whom. This explains why the name of some of the initial codes in this coding tree do not, on first sight, fit the related category or larger domain. The re-arrangements were discussed by first coder MK and first author MH, and also within the peer group of the other authors. The initial codebook (in Dutch) is available on request.

**Domains**	**Categories**	**Codes**
1. The family caregiver	Facilitator: Comics in general being easily accessible	• Form of the book: remarks about accessibility<br>• Content of the book: remarks about accessibility<br>• Facilitating factor: absence of (difficult) language<br>• Impact of the book: the book evokes feelings*<br>• Content of the book: images have more impact than text<br>• Actual use of the book: importance of examining and 'sensing' the fit with the family caregiver*<br>• Opinions about (hypothetical) target audiences<br>○ Background of the family caregiver<br>○ Migrant family caregivers<br>○ The book has no language barrier<br>○ People with low literacy skills
	Barrier: Misfit with the specific person, phase, or setting	• Actual use of the book: importance of 'sensing' the fit with the family caregiver*<br>• Facilitating factors:<br>○ Similar setting

		○ Story of the family caregiver shows similarities with story of the book<br>• Hindering factors:<br>○ Lack of knowledge about the family caregiver's situation<br>○ Inappropriate setting or type of relationship<br>○ Insecurities about the impact of the book<br>• Opinions about (hypothetical) target audiences<br>○ The fit with the phase of the illness of the care receiver<br>○ Family caregiving in a non-palliative phase<br>○ People with a chronic illness<br>○ People with a mental illness<br>• Actual use of the book: remarks about using separate images without context*<br>• Content of the book: remarks about metaphors
2. Impact on the family caregiver	Facilitator: The book being recognizable and supportive, and raising awareness about family care	• Positive impact of the book<br>○ The book evokes associations in the family caregiver<br>○ The book provides recognition for the family caregiver<br>○ The book is recognizable*<br>○ The book touches emotionally*<br>○ The book evokes feelings*<br>○ The book is supportive<br>○ The book provides bereavement support<br>○ The book raises awareness among family caregivers to ask for help<br>○ Reading the book leads to action<br>○ Reading the book leads to accepting help of others<br>• Actual use of the book: conversation with help of the book increases recognition of the family caregiver
	Barriers: The book being too confronting and not supportive in conversations	• Negative impact of the book<br>○ The book is harsh, confronting, shocking, too direct<br>○ The book is depressing, gloomy, negative<br>○ The book evokes resistance/aversion in the family caregiver<br>• Negative aspects of the content of the book

		○ The black-and-white storyline is not representative, too negative, unloving*<br>○ Absence of beautiful moments in family caregiving*<br>• Hindering factors:<br>○ The supporter wants to protect the family caregiver from becoming overburdened<br>○ The dark storyline is confronting<br>○ Insecurities about the impact of the book*<br>○ The book is not recognizable
3. Impact on the conversation between the person who provides support and the family caregiver	Facilitators: Raising specific conversation topics, deepening the conversation	• The book is helpful as a conversation aid<br>• Facilitating factor: the book helps in keeping the focus of the conversation on certain topics
	Facilitators: Raising awareness among people who provide support in palliative care, evoking specific questions	• Positive impact of the book<br>○ Raises awareness among professional caregivers<br>○ Raises awareness among volunteers<br>○ Raises awareness among healthcare students<br>○ Raises awareness among people surrounding the family caregiver<br>○ Colleagues share their own experiences with each other via the book<br>• Opinions about (hypothetical) target audiences: people surrounding the family caregiver
	Barriers: Being too directive and having no surplus-value with regard to topics being discussed	• Hindering factors<br>○ The book is too directive and unnatural<br>○ The book is not useful for the conversation<br>• The book is not helpful as a conversation aid<br>• Negative impact of the book: the book has no surplus-value
4. Relationship between the person who provides support and the family caregiver	Facilitator:<br>Existing relationship with the family caregiver, possibility of follow-up	• Actual use of the book: importance of examining and 'sensing' the fit with the family caregiver*<br>• Facilitating factors:<br>○ Knowing the family caregiver well*<br>○ Importance of trust in the professional caregiver or volunteer

		○ Time<br>• Actual use of the book: importance of follow-up
	Short-time contact, risk of damaging relationship or image as a professional	• Hindering factors:<br>○ Time<br>○ The book goes ‘too deep’ for first contact<br>○ Insecurities about follow-up<br>○ Risk of damaging trust and relationship<br>• Negative impact of the book: the book raises resistance/aversion in the person who provides support
5. Person who provides support to family caregivers within palliative care	Methods of presenting and using the book in conversations with family caregivers (NB: as elaborated in the introducing paragraph of the Results section)	• Actual introduction of the book<br>• Actual use of the book<br>• Methods of using the book
	Opinions about the content and form of the book (NB: as elaborated in the introducing paragraph of the Results section)	• Positive impact of the book<br>○ The book is recognizable*<br>○ The book touches emotionally*<br>○ The book evokes feelings*<br>• Positive aspects of the content of the book<br>○ Beautiful<br>○ Shows beautiful moments in family caregiving<br>○ Colored storyline and the tender loving care this storyline shows<br>○ Positive remarks<br>○ Title<br>○ Complementary storylines<br>• Negative aspects of the content of the book<br>○ The black-and-white storyline is not representative, too negative, unloving*<br>○ Absence of beautiful moments in family caregiving<br>○ Complicated because of the storylines being mixed together<br>○ The aim of the book is unclear<br>○ The book is unclear

		○ The book is 'too much' or too thick<br>○ The book lacks information<br>• Content of the book: remarks about specific scenes or themes<br>• Content of the book: remarks about the black-and-white storylines<br>• Form of the book: preference for text<br>• Hindering factor: the storylines being mixed together<br>• Needs of the participants:<br>○ The book should have shown more beautiful things and more light-heartedness<br>○ The black-and-white storylines should have been less dark<br>○ The book should have shown certain themes<br>○ More information needed about definition of family care and aim of the book<br>○ More text and explanation needed<br>○ Index of the book needed<br>○ Guide for using the book needed<br>○ Separation of the two storylines<br>○ Separate images needed<br>○ Separate images without text needed<br>○ A second book is needed
	Facilitator: Enthusiasm about, access to, and familiarity with Naasten	• Facilitating factors:<br>○ Having your own enthusiasm and curiosity regarding the book's possible value<br>○ Knowing the book well<br>○ Having the book's themes in the back of your head<br>○ Always carrying the book with you<br>○ Own experiences with family caregiving<br>○ Having experience with using the book<br>○ "Just do it"
	Barrier: Unfamiliarity with the comics medium	• Hindering factors:<br>○ Not needing the book as a conversation aid<br>○ Having more trust in one's own familiar conversational techniques than in this medium

	Target audiences for the book (supporters who could benefit from reading the book) (NB: as elaborated in section B of the Results section, about the novel's potential value for education)	• Target audiences<br>○ People who provide support: professional caregiver<br>○ People who provide support: volunteer<br>○ Healthcare students or professionals in training<br>○ Importance of age<br>• Positive impact of the book<br>○ Raises awareness among professional caregivers*<br>○ Raises awareness among volunteers*<br>○ Raises awareness among healthcare students*<br>○ Raises awareness among people surrounding the family caregiver*<br>• Suggestions of the participants: using the book in education<br>• Hypothetical methods of using the book

*Several codes fitted multiple facilitators or barriers and, hence, are reported multiple times in this coding tree.
