# Supplementary material for: Facilitators and barriers in using comics to support family caregivers of patients receiving palliative care at home: A qualitative study
Source: Palliat Med. 2022 May 3;36(6):994–1005. doi: 10.1177/02692163221093513 (PMC9174613; doi:10.1177/02692163221093513)
Supplement: sj-pdf-6-pmj-10.1177_02692163221093513 – Supplemental material for Facilitators and barriers in using comics to support family caregivers of patients receiving palliative care at home: A qualitative study [file sj-pdf-6-pmj-10.1177_02692163221093513.pdf]

Supplemental file 1
Title: Examples of pages from the graphic novel *Naasten* about family caregiving at home

Description: This file shows several pages from the Dutch graphic novel *Naasten* (English: Loved ones). This 230-page book tells the stories of two characters caring for their partner or family member receiving palliative care at home. It was based on themes and scenes from the qualitative interview study that was part of our larger research project. The images in this file provide context for the results of the current study about the graphic novel's value as a conversation aid to support family caregivers, and as a tool for educating professionals and volunteers.

Supplemental file 2
Title: Topic guide for our focus groups with professionals and volunteers who supported family caregivers
(No additional description needed.)

Supplemental file 3
Title: Topic guide for our telephone interviews with family caregivers
(No additional description needed.)

Supplemental file 4
Title: COREQ checklist for this study
(No additional description needed.)

Supplemental file 5
Title: Coding tree
(No additional description needed. We have provided an explanation in the file itself.)
